# Matrix metalloproteinases and their inhibitors in human traumatic spinal cord injury

**DOI:** 10.1186/1471-2377-7-17

**Published:** 2007-06-26

**Authors:** Armin Buss, Katrin Pech, Byron A Kakulas, Didier Martin, Jean Schoenen, Johannes Noth, Gary A Brook

**Affiliations:** 1 Department of Neurology, Aachen University Hospital, Aachen, Germany; 2 Centre for Neuromuscular and Neurological Disorders, University of Western Australia, Perth, Australia; 3 Department of Neurosurgery, Sart Tilman Hospital, University of Liège, Liège, Belgium; 4 Departments of Neurology and Neuropathology, University of Liège, Liège, Belgium; 5 Department of Neuropathology, Aachen University Hospital, Aachen, Germany

## Abstract

**Background:**

Matrix metalloproteinases (MMPs) are a family of extracellular endopeptidases that degrade the extracellular matrix and other extracellular proteins. Studies in experimental animals demonstrate that MMPs play a number of roles in the detrimental as well as in the beneficial events after spinal cord injury (SCI). In the present correlative investigation, the expression pattern of several MMPs and their inhibitors has been investigated in the human spinal cord.

**Methods:**

An immunohistochemical investigation in *post mortem *samples of control and lesioned human spinal cords was performed. All patients with traumatic SCI had been clinically diagnosed as having "complete" injuries and presented lesions of the maceration type.

**Results:**

In the unlesioned human spinal cord, MMP and TIMP immunoreactivity was scarce. After traumatic SCI, a lesion-induced bi-phasic pattern of raised MMP-1 levels could be found with an early up-regulation in macrophages within the lesion epicentre and a later induction in peri-lesional activated astrocytes. There was an early and brief induction of MMP-2 at the lesion core in macrophages. MMP-9 and -12 expression peaked at 24 days after injury and both molecules were mostly expressed in macrophages at the lesion epicentre. Whereas MMP-9 levels rose progressively from 1 week to 3 weeks, there was an isolated peak of MMP-12 expression at 24 days. The post-traumatic distribution of the MMP inhibitors TIMP-1, -2 and -3 was limited. Only occasional TIMP immuno-positive macrophages could be detected at short survival times. The only clear induction was detected for TIMP-3 at survival times of 8 months and 1 year in peri-lesional activated astrocytes.

**Conclusion:**

The involvement of MMP-1, -2, -9 and -12 has been demonstrated in the post-traumatic events after human SCI. With an expression pattern corresponding largely to prior experimental studies, they were mainly expressed during the first weeks after injury and were most likely involved in the destructive inflammatory events of protein breakdown and phagocytosis carried out by infiltrating neutrophils and macrophages, as well as being involved in enhanced permeability of the blood spinal cord barrier. Similar to animal investigations, the strong induction of MMPs was not accompanied by an expression of their inhibitors, allowing these proteins to exert their effects in the lesioned spinal cord.

## Background

After traumatic spinal cord injury (SCI), the initial damage to the parenchyma at the lesion site is followed by a complex cascade of secondary events including breakdown of the blood spinal cord barrier (BSB) and infiltration of blood-derived inflammatory cells, oedema, excitotoxicity and ischemia. Experimental investigations have revealed that this phase of secondary parenchymal damage spans the first 48–72 hours post injury and is followed by the removal of tissue debris over subsequent weeks. Finally, the lesion site of severely injured tissue becomes dominated by scar tissue comprised of connective tissue, including Schwann cells and meningeal fibroblasts, and fluid-filled cysts, surrounded by a dense astroglial scar [1]. Remodelling of the extracellular matrix (ECM) plays an important role in most of these events

Matrix metalloproteinases (MMPs) are a family of extracellular zinc- and calcium-dependent endopeptidases that degrade the extracellular matrix and other extracellular proteins [2]. The 23 mammalian MMPs can be placed into sub-groups based on structural similarities and substrate specificity and they are capable of degrading virtually all extracellular proteins. Once activated, MMPs are subject to inhibition by 4 different tissue inhibitors of metalloproteinases (TIMPs) that bind MMPs non-covalently [3]. MMPs are involved in events requiring matrix remodelling in developmental processes, wound healing and repair throughout life. In the nervous system, these enzymes play a role in the migration of precursor cells to their destination and are directly involved in axonal outgrowth during development [4]. However, the aberrant expression of members of this protein family is involved in disease processes such as cancer metastasis and CNS disorders including multiple sclerosis, stroke, Alzheimer's disease and trauma [4,5]. Recent experimental SCI investigations have demonstrated an involvement of MMPs in the post-traumatic events. In a mouse spinal cord compression injury model, a significant up-regulation of 11 MMPs was demonstrated [6]. Most of the proteins, including MMP-3, -9 and -10, showed an early induction, starting 24 hours after injury whereas the expression of other MMPs, including MMP-2, -12 and -13, was delayed until 5 days after trauma. Further studies into the role of MMP-12, the most markedly up-regulated MMP, demonstrated improved recovery in MMP-12 null mice, most likely due to a reduction of the lesion-induced permeability of the blood spinal cord barrier (BSB) and a reduced density of microglia/macrophages at the lesion site [6]. An investigation into the role of MMP-9 in SCI showed similar results in a contusion injury model, with improved locomotor function in MMP-9 null mice as compared to wild-type animals. Again, a reduction of the lesion-induced permeability of the BSB and a resulting attenuation of inflammatory cell infiltration were suggested to be the likely mechanisms for the favourable outcome [7]. Another study in mice undergoing a spinal cord contusion injury correlated the up-regulation of MMP-2 mainly to the formation of the glial scar [8]. In MMP-2 null mice, there was reduced white matter sparing, less nerve fibre regeneration and a more extensive astroglial scarring compared to their wild type littermates. These results suggest that MMP-2 up-regulation promotes functional recovery after SCI by regulating glial scar formation and white matter sparing.

Therefore, studies in experimental animals have demonstrated that MMPs play a number of roles in the detrimental, early inflammation-related processes as well as in the later, beneficial wound healing and regenerative events [4,5]. In the present investigation, the expression pattern of several MMPs and their inhibitors has been investigated in samples of *post mortem *human spinal cord, taken from 15 patients who died at a range of survival times following traumatic SCI. Our data reveals an induction of MMPs-1, -2, -9 and -12 with an individual profile for each protein. The present data demonstrates a strong correlation with some aspects of the data obtained from experimental animals. However, some clear differences appear to exist between animal and human tissues following spinal cord injury.

## Methods

*Post mortem*, the spinal cords were removed from 4 control patients who had not suffered from any neurological disease (Table 1) and from 15 patients who died at a range of time points after traumatic spinal cord injury (Table 2). The study was approved by the Aachen University Ethics Committee. Patients with traumatic injury had been diagnosed as having "complete" injuries and presented with paraplegia or tetraplegia. The spinal columns of both control and SCI patients were removed at autopsy, approximately 15–48 hours after death. Following incision of the dura mater, the spinal cord was fixed in 4% buffered formaldehyde for at least 2 weeks. Thereafter, blocks of the lesion site and/or tissue from regions rostral and caudal to the lesion (approximately 1 cm thickness) were embedded in paraffin wax.

**Table 1:** Patients who served as the control group

**Case number**	**Age**	**Cause of death**
1	29 years	Breast cancer
2	47 years	Pneumonia
3	62 years	Breast cancer
4	83 years	Myocardial infarction

**Table 2:** Patients who died after traumatic injury to the spinal cord

**Case number**	**Age**	**Injury level**	**Inj.-death interval**
1	21 years	T12	2 days
2	51 years	C1	4 days
3	84 years	C3-4	5 days
4	65 years	C5	8 days
5	63 years	C6	10 days
6	18 years	T6	11 days
7	72 years	T11-12	24 days
8	85 years	C3	4 months
9	76 years	T8-9	10 months
10	80 years	C5-6	1 year
11	72 years	T12	2 years
12	44 years	L1	8 years
13	71 years	C3-4	20 years
14	47 years	T5	26 years
15	57 years	T3-4	30 years

### Peroxidase immunohistochemistry

Transverse sections (5 μm thick) were collected onto poly-L-lysine-coated slides and allowed to dry. Sections were de-waxed in xylene and rehydrated. Microwave treatment in 10 mM citrate buffer (pH 6) for 3 × 3 minutes was followed by blockade of non-specific binding by incubation in 0.1 M PBS containing either 3% normal horse or normal goat serum and 0.5% triton X-100 for 30 minutes. Sections were subsequently incubated in the following primary antibodies, overnight at room temperature: mouse monoclonal anti-MMP-1 (1:4000; Chemicon), mouse monoclonal anti-MMP-2 (1:20; Calbiochem), mouse monoclonal anti-MMP-9 (1:100; R&D Systems), mouse monoclonal anti-MMP-12 (1:10; R&D Systems), mouse monoclonal anti-TIMP-1 (1:300; Chemicon), rabbit polyclonal anti-TIMP-2 (1:250; Chemicon), mouse monoclonal anti-TIMP-3 (1:100; Chemicon), rabbit polyclonal anti-glial fibrillary acidic protein (1:2500, DAKO) and mouse monoclonal anti-CD68 (1:50; DAKO). Following extensive rinsing steps in 0.1 M PBS, sections were incubated in biotinylated horse anti-mouse or goat anti-rabbit antibody (diluted 1:500, Vector Laboratories) for 1 hour at room temperature. Incubation with the biotinylated secondary antibody was followed by the Vector ABC system and a subsequent incubation in diaminobenzidine for visualization of the reaction product. For negative controls the primary antibody was omitted. Those sections incubated with the CD68 antibody were finally counterstained with 0.2% thionin for 1 minute.

### Immunofluorescence

Sections were de-waxed in xylene and rehydrated. Microwave treatment in 10 mM citrate buffer (pH 6) for 3 × 3 minutes was followed by blockade of non-specific binding by incubation in 3% normal goat serum in 0.5% triton X-100 in 0.1 M PBS for 30 minutes and subsequent incubation overnight at room temperature with the anti-MMP-1 antibody (1:250) or the anti-TIMP-3 antibody (1:20) and the anti-GFAP antibody (1:200). Following incubation with Alexa 594 (red-fluorescence)-conjugated goat anti-mouse and Alexa 488 (green fluorescence)-conjugated goat anti-rabbit secondary antibodies (diluted 1:500, Molecular Probes) for 3 hours at room temperature, slides were cover-slipped in Moviol.

For MMP and CD68 double staining, the tyramide signal amplification kit (TSA Cyanine 3 system, NEL704A, PerkinElmer Life Sciences) was used. Briefly, following the blockade of endogenous tissue peroxidase, sections were rinsed in the TSA block buffer (prepared as recommended by the manufacturers) and incubated with the anti-MMP-1, -2, -9 or -12 antibodies at a dilution of 1:200 overnight. Incubation with a biotinylated horse anti-mouse antibody (1:500, BA2000, Vector) for 1 hour, rinsing in 0.05% Tween20 and blocking with the provided reagent for 30 minutes was followed by streptavidin-HRP (1:500) in blocking reagent for 30 minutes and Cyanine 3-tyramide working solution (1:100) for 10 minutes. After rinsing, the slides were incubated with the monoclonal anti-CD68 primary antibody (1:30) overnight, followed by a Cy2-conjugated goat anti-mouse antibody (1:100, Jackson Laboratories) for 3 hours at room temperature. Finally, nuclei were stained for 5 minutes with DAPI (diluted 1:1000, Sigma) and the sections were cover-slipped with Moviol. For negative controls, the primary antibodies were omitted.

For a semi-quantitative description of the amount of MMP-immunoreactive cells at the various survival times, an arbitrary rating scale for the number of labelled cells was chosen, ranging from 0 to ++++. A value of 0 indicated the detection of no immunopositive cells while a value of ++++ indicated the presence of large numbers of labelled cells.

## Results

Samples of spinal cord from all 19 cases were examined using immunohistochemistry for MMP-1, -2, -9 and -12, TIMP-1, -2 and -3 as well as CD68 and GFAP. The brains of all cases were carefully examined and were declared to be without pathological findings. The spinal cords of the control cases were also morphologically intact. For an overview of the results, especially the number of immunoreactive cells at the lesion epicentre at the various survival times, see table 3. The interpretation of the semi-quantitative presentation of MMP and TIMP expression is restricted, due to the limited comparability of staining quality (largely due to differences in the time between death and the dissection/fixation of tissue). However, immunohistochemistry was performed using the identical staining procedure and cases with closely matching survival times (e.g. 4–5 days and 10–11 days after SCI) demonstrated very similar amounts of MMP and TIMP immunoreactivity.

**Table 3:** Amount of immunopositive cells at the lesion epicentre at different survival times following human SCI

	**Control**	**2 days**	**4–5 days**	**8 days**	**10–11 days**	**24 days**	**4 months**
**CD68**	0/+	+	+++	++++	++++	++++	++
**MMP-1**	+	+	++	+++	+	+	+ (astrocytes)
**MMP-2**	0	+	++	+++	++	+	0
**MMP-9**	0/+	+	++	+++	+++	+++	0/+
**MMP-12**	0	+	+	+	+	++++	0
**TIMP-1**	0	0/+	0/+	0/+	0/+	0/+	0
**TIMP-2**	+	0/+	0/+	0/+	0/+	0/+	+
**TIMP-3**	0/+	0/+	0/+	0/+	0/+	0/+	0/+

The figures reflect the number of immunopositive cells using an arbitrary rating scale from 0 (no cells) to ++++ (maximum amount of cells) in sections from control spinal cords and the lesion core at various survival times after SCI stained for the different antigens.

### Normal distribution of MMPs and TIMPs in the human spinal cord

**Figure 1:**
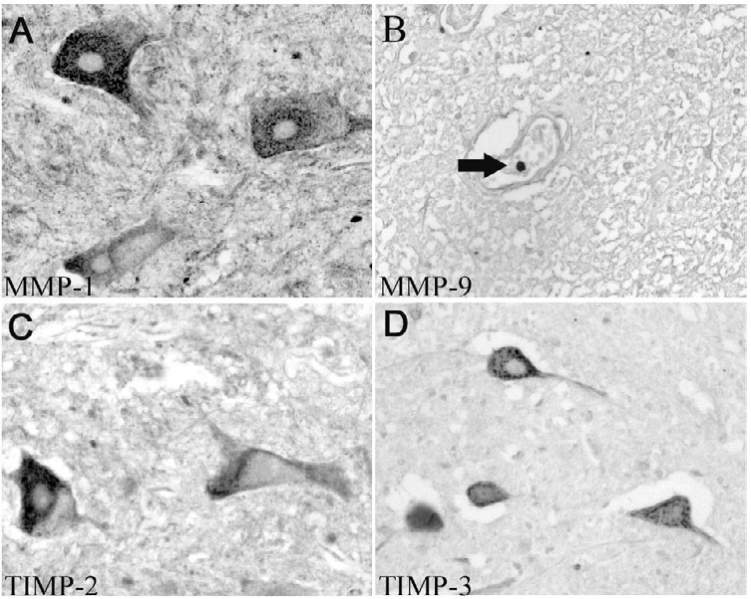
**Distribution of MMPs and TIMPs in the normal human spinal cord**. Images were taken from control human spinal cords in transverse sections. **A: **Immunohistochemistry for MMP-1 revealed cytoplasmic staining in motoneurons; the nucleus was devoid of immunoreactivity. **B: **Staining for MMP-9 demonstrated a single intravascular monocyte (arrow) within a parenchymal blood vessel. **C: **The TIMP-2 antibody displayed cytoplasmic immunoreactivity in motoneurons, the nucleus being devoid of staining. **D: **Immunohistochemistry for TIMP-3 also shows cytoplasmic staining in motoneurons without a nuclear signal. **(A-D, mag. ×260)**

The spinal cord parenchyma including the meninges was immunonegative for MMP-2 and -12 and TIMP-1 (not shown). Immunohistochemistry for MMP-1 revealed staining in most motoneurons and Clarke's nucleus neurons as well as scattered interneurons, mostly in laminae IV to VI (Fig.1A). Staining for MMP-9 demonstrated rare intravascular monocytes in both meningeal and parenchymal blood vessels (Fig.1B). TIMP-2 immunoreactivity was present in most motoneurons and individual Clarke's nucleus neurons and interneurons from laminae I to VI (Fig.1C). TIMP-3 revealed a similar distribution but with only approximately 50% of motoneurons stained in cervical, thoracic and lumbar segments (Fig.1D). This distribution was detected in all 4 control cases, and no evidence for age-related changes in the distribution of MMP and TIMP isoforms could be found in any of the normal human CNS samples.

### Macrophage/microglial responses to human SCI

**Figure 2:**
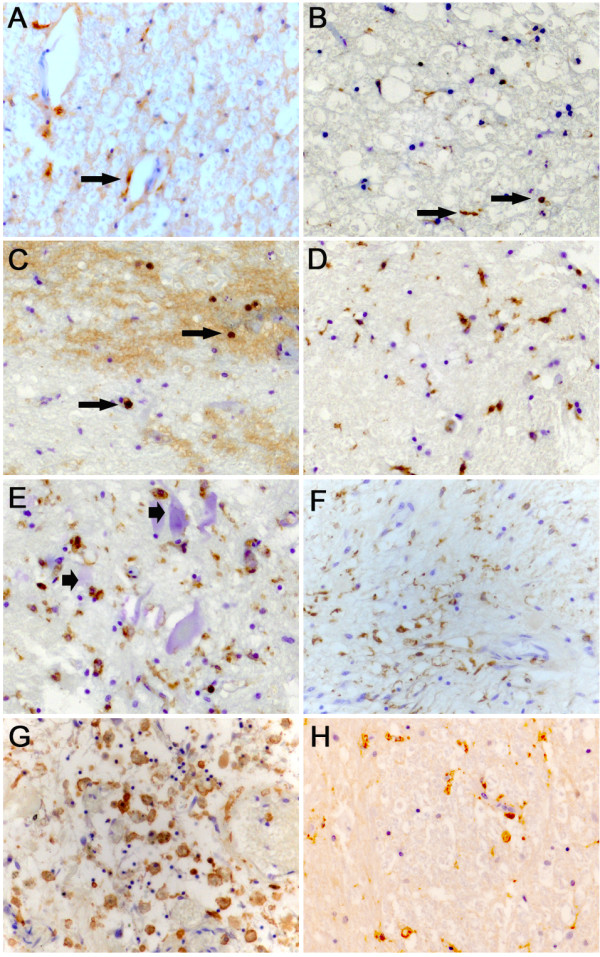
**Macrophages/microglia in the human spinal cord after traumatic injury**. Images were taken from control spinal cords and cases with different survival times after SCI in transverse sections stained with the CD68 antibody. **A: **In a control spinal cord, CD68 immunohistochemistry reveals bipolar peri-vascular cells (arrow). **B: **Two days after SCI, sections from the lesion site demonstrated a clear up-regulation of CD68-positive cells. Round to oval shaped cells (arrows) were spread over the area. **C: **In the same case, areas of bleeding contained some round immunopositive macrophages (arrows). The diffuse brown background staining was unspecific, being due to the high concentration of endogenous peroxidase in the blood cells. **D: **4 days after injury, the lesion area displayed an increased number of CD68-positive cells, many with activated microglial and rounded macrophage-like morphologies. **E: **In the peri-lesional gray matter of the same case, identifiable neurons (arrowheads) were often closely surrounded by CD68 positive cells. **F: **10 days after injury, the lesion epicentre was filled with numerous microglia/macrophages. **G: **24 days after injury, the lesion epicentre was heavily loaded with large, foamy macrophages. **H: **Four months after SCI, only few immunoreactive cells can be seen at the site of injury. **(A-H, mag. ×260)**

In the normal unlesioned spinal cord, CD68 immunoreactivity was scarce. Occasional bipolar perivascular cells (e.g. arrowheads, Fig.2A) and intravascular monocytes were immunopositive. Although sections from the lesion site demonstrated no preservation of cytoarchitecture at 2 days after SCI, there was a slight increase in the incidence of CD68-positive cells. These cells were round to oval-shaped and were spread over the lesion core and the peri-lesional area (Fig.2B–C). By 4 and 5 days after injury, the number of immunopositive cells at the lesion epicentre was further increased. Microglia/macrophages could be found in the area of necrosis, either as individual cells or as small clusters (Fig.2D). In peri-lesional gray matter areas, reactive CD68-positive microglia/macrophages were sometimes found in close apposition to neuronal cell bodies (Fig.2E). By 8, 10, 11 and 24 days post injury, the lesion core contained numerous microglia/macrophages, mostly with the morphology of foamy macrophages (Fig.2F and 2G) but by 4 months, fewer CD68 immunoreactive cells could be detected at the lesion site (Fig.2H). Sections from tissue blocks of all later survival times demonstrated a few remaining immunoreactive cells with a peri-vascular distribution, similar to that seen in the control cases (not shown).

### Astrocytic reaction to human SCI

**Figure 3:**
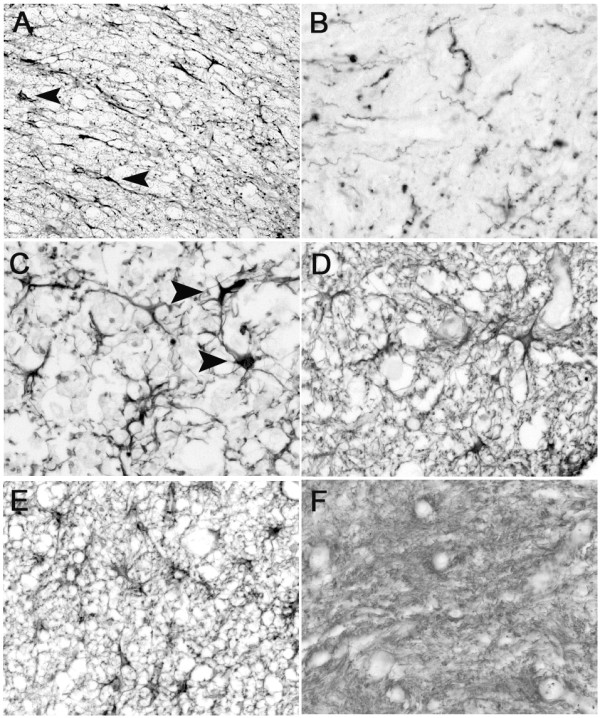
**Astrocytes in the human spinal cord after traumatic injury**. Images were taken from control spinal cords and cases with different survival times after SCI in transverse sections stained with the GFAP antibody. **A: **GFAP immunohistochemistry in the unlesioned spinal cord white matter revealed astrocytic cell bodies (arrowheads) in between a homogenous network of processes. **B: **At 2 days after trauma, areas of massive tissue destruction demonstrated a dramatic loss of GFAP immunoreactivity. Hardly any astroglial cell bodies were visible and an irregular loose arrangement of processes can be seen. **C: **8 days after injury, highly GFAP-positive activated astrocytes were spread over peri-lesional white and gray matter (arrowheads) with an increased density of processes. **D: **Eleven days after SCI, large, highly GFAP-positive astrocytes were surrounded by a dense irregular network of processes. **E: **In the peri-lesional area, about 1 segment away from the lesion site, activated astroglia could be detected 1 year after trauma. **F: **At the lesion site of the same case, a dense astroglial GFAP-positive scar was visible in which the cell bodies were hardly detectable. **(A-F, mag. 215;260)**

GFAP immunohistochemistry in the normal, unlesioned spinal cord revealed astrocytic cell bodies evenly dispersed throughout spinal cord gray and white matter, scattered between the network of astrocytic processes (Fig.3A). At early survival times, spanning 2 to 11 days after trauma, the areas of massive tissue destruction demonstrated a massive reduction of GFAP immunoreactivity. Hardly any astroglial cell bodies and an irregular, loose arrangement of processes were detectable (Fig.3B). In cases with survival times of 4 days and longer, perilesional, highly GFAP-positive, activated astrocytes could be seen. These cells were distributed homogeneously over white and gray matter in sections up to 1 to 2 segments proximal and distal to the lesion site (Fig.3C). By eleven days, nests of large, intensely GFAP-positive astrocytes surrounded by a dense irregular network of processes could be observed (Fig.3D). The formation of the glial scar progressed, and in cases with survival times of 4 months and longer, a dense astroglial, GFAP-positive, scar was visible in which the cell bodies were hardly detectable (Fig.3E). In the peri-lesional areas up to 1 segment away from the lesion site, activated astroglia could still be detected up to 1 year after trauma (Fig.3F).

### Expression of MMP-1 after human SCI

**Figure 4:**
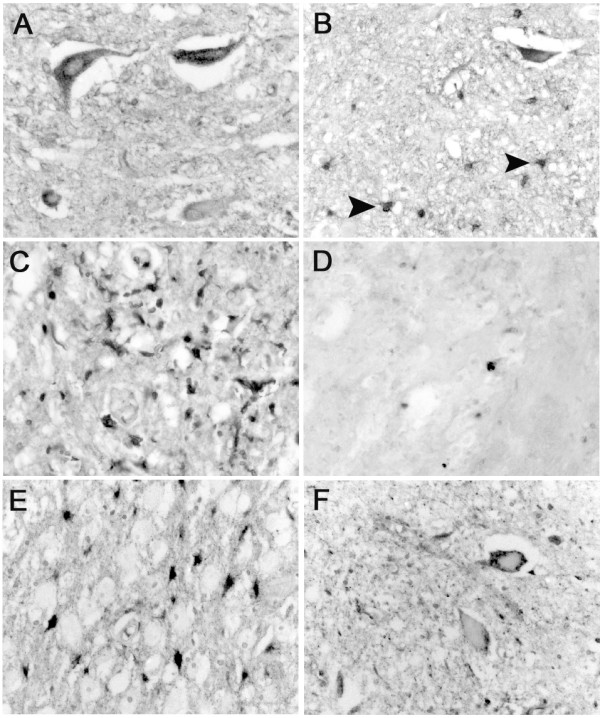
**MMP-1 in the human spinal cord after traumatic injury**. Images were taken from cases with different survival times after SCI in transverse sections stained with the MMP-1 antibody. **A: **Two days after injury, motoneurons in the perilesional area retained their cytoplasmic MMP-1 immunoreactivity. **B: **Four days after SCI, MMP-1 immunohistochemistry at the site of injury displayed round to oval cells (arrowheads). **C: **By 8 days, the lesion site was filled with a high number of MMP-1 immunoreactive cells. Again, more round to oval shaped cells can be seen. **D: **At 11 days after SCI, the amount of MMP-1 stained cells was dramatically decreased and only single round cells were detectable. **E: **4 months after injury, large MMP-1 positive cells could be seen around the site of injury with the morphology of activated astrocytes. **F: **8 months after SCI, identifiable neurons around the lesion site demonstrated a staining pattern similar to that of control cases. **(A-F, mag. ×260)**

**Figure 7:**
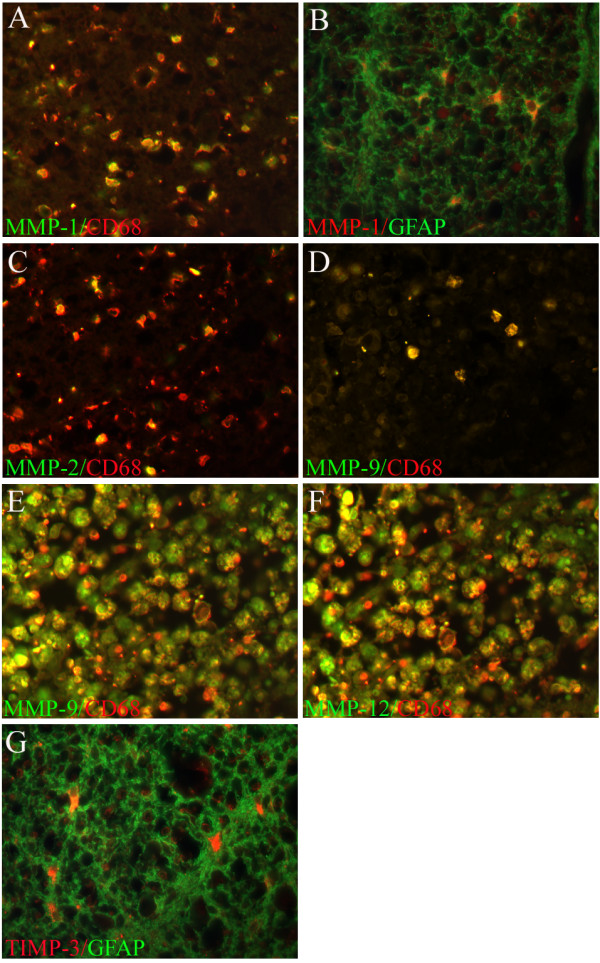
**MMP-1, -2, -9 and -12 and TIMP-3 in macrophages and astrocytes after traumatic human spinal cord injury**. Images were taken from cases with different survival times after SCI in transverse sections.**A: **8 days after injury. Double immunofluorescence for CD68 (red) and MMP-1 (green). Almost all microglia/macrophages were MMP-1 positive at the lesion site. **B: **8 months after injury. Double immunofluorescence for GFAP (green) and MMP-1 (red). Large, activated, strongly GFAP-positive astrocytes expressing MMP-1 were observed in the glial scar tissue. **C: **11 days after trauma, MMP-2 immunoreactivity (green) was expressed by CD68 positive microglia/macrophages (red) at the lesion site. **D: **2 days after injury, CD68 positive microglia/macrophages (red) were MMP-9 immunoreactive (green) at the lesion epicentre. **E: **24 days after trauma, dense packing of large, CD68 positive macrophages (red) which also stained for MMP-9 (green) was visible at the lesion epicentre. **F: **In the same case, double immunofluorescence with CD68 (red) and MMP-12 (green) showed a nearly identical distribution. **G: **Eight months after injury, activated GFAP-positive astrocytes (green) displayed TIMP-3 immunoreactivity (red) in the perilesional scar. **(A-G, mag. ×400)**

At all survival times, the neuronal distribution of MMP-1 in the peri-lesional area remained unchanged (Fig.4A and 4F). At 2 days, some round to oval cells were seen at the site of injury. They were detectable in the heavily destroyed tissue but were present at a lower density than the CD68- positive macrophages/microglia. Four days after trauma, immunoreactive cells, morphologically resembling macrophages, could be seen in areas of bleeding and massive tissue destruction (Fig. 4B) and by 8 days, the lesion core contained substantial numbers of immunoreactive cells, corresponding in size, distribution and morphology to the CD68 (and MMP-9, see below)-positive cells observed in near adjacent sections (Fig.4C and 7A). By 10–24 days, the incidence of MMP-1 positive cells was dramatically decreased and only single round cells were detectable (Fig.4D). However, sections taken from the peri-lesional area between 4 months and 1 year post injury revealed large, MMP-1 positive cell bodies which were subsequently demonstrated to be astrocytes by double immunofluorescence (Fig.4E and 7B).

### Expression of MMP-2 after human SCI

**Figure 5:**
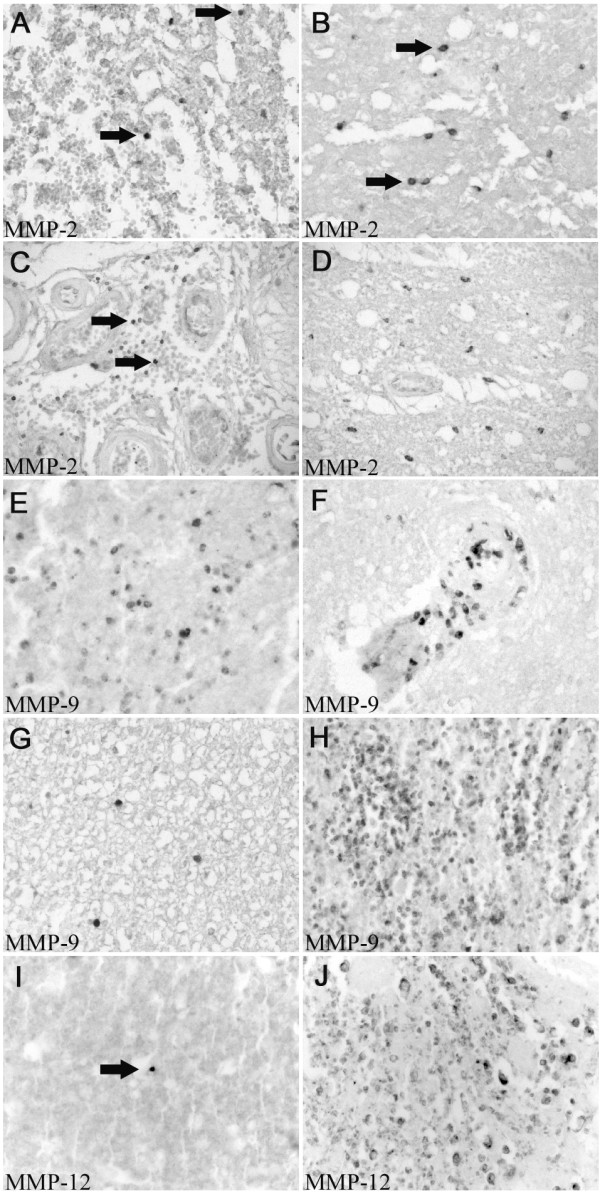
**MMP-2, -9 and -12 in the human spinal cord after traumatic injury**. Images were taken from cases with different survival times after SCI in transverse sections. **A: **Immunohistochemistry for MMP-2 two days after injury. A few positive round cells (arrows) in areas of massive tissue destruction and hemorrhagic infiltration could be seen. **B: **In the same case, areas of non-haemorrhagic lesion site revealed a higher number of immunoreactive cells with a round to oval morphology (arrows). **C: **At 8 days after SCI, MMP-2 immunoreactive microglia/macrophages were still visible in areas of bleeding (arrows). **D: **In the same case, the peri-lesional area demonstrated round to oval cells (arrows) with a density and distribution corresponding to earlier survival times. **E: **Two days after traumatic SCI, staining for MMP-9 demonstrated immunoreactive cells (arrows) with a mostly rounded morphology, in areas close to the lesion but without signs of hemorrhagic infiltration. **F: **In contrast to the more even distribution in **E**, clusters of positive cells could be seen in and around blood vessels (arrow). **G: **In sections one segment away from the lesion, hardly any MMP-9 positive cells were detectable. **H: **At 24 days after injury, large numbers of MMP-9 positive round cells were visible at the lesion epicentre, filling the area of tissue destruction. **I: **At a survival time of 2 days, immunohistochemistry for MMP-12 displayed rare positive cells (arrow) at the lesion site with a round morphology. **J: **At 24 days after SCI, many large round to oval shaped cells demonstrated strong MMP-12 immunoreactivity at the site of injury. **(A-J, mag. ×260)**

Immunohistochemistry for MMP-2 demonstrated the first positive cells in sections from the lesion epicentre at 2 days after injury. In areas of massive destruction and hemorrhagic infiltration, a few positive round cells were detected whereas areas of the lesion site without bleeding demonstrated a higher number of immunoreactive cells with a round to oval morphology (Fig.5A–B). By 8 days, the morphology and distribution of the cells within the lesion site corresponded to those of CD68-positive macrophages (Fig.5C–D). However, by 10 days the number of MMP-2 positive macrophages was clearly reduced (Fig.7C) and by 24 days, only single immunoreactive cells could be detected. At all later survival times, no specific staining was visible, similar to control cases (not shown).

### Expression of MMP-9 after human SCI

By 2 days after injury (the earliest time point investigated) staining for MMP-9 demonstrated immunoreactive cells at and around the lesion site. In areas of severe tissue destruction and intensive bleeding, only few cells with a round morphology were stained (Fig.5E). At 4–5 days after injury, in areas at the lesion epicentre but without signs of hemorrhagic infiltration, the amount of immunoreactive cells was higher compared to other heavily destroyed areas. The cells were mostly of a round morphology and were relatively evenly distributed except for clusters of positive cells in and around blood vessels (Fig.5F). Double immunofluorescence with an anti-CD68 antibody demonstrated a microglia/macrophage origin of most MMP-9 positive cells (Fig.7D). Morphologically, the MMP-9 positive/CD68-negative cells at these early survival times resembled infiltrating neutrophils. In sections further away from the lesion, the number of immunoreactive cells decreased rapidly; such that hardly any MMP-9 positive cell could be detected one segment distal or proximal of the site of injury (Fig.5G). This distribution remained more or less constant up to 11 day post injury. By 24 days, large numbers of MMP-9 positive round cells were visible at the lesion core and filled the area of tissue destruction (Fig.5H). Double immunofluorescence revealed these cells to be phagocytosing macrophages (Fig.7E). At survival times of 4 months and later, only single MMP-9 immunoreactive cells were detectable at and around the lesion site, showing a distribution similar to that of control cases (not shown). Apart from microglia/macrophages and some neutrophils, no other cell population demonstrated immunoreactivity for MMP-9 at all time points (not shown).

### Expression of MMP-12 after human SCI

In contrast to the other MMPs investigated, immunohistochemistry for MMP-12 only displayed rare immunopositive, rounded cells at and around the lesion site up to 11 days post injury (Fig.5I). This distribution changed dramatically by 24 days, when many intensely immunoreactive microglia/macrophages could be detected at the lesion core (Fig.5J and 7F). The number of stained cells decreased rapidly in sections further away from the lesion such that by 1 segment proximal and distal no immunoreactive cells were visible. In all cases with longer survival times, no specific MMP-12 staining was seen at and around the lesion site (not shown).

### Expression of TIMP-1, -2 and -3 after human SCI

**Figure 6:**
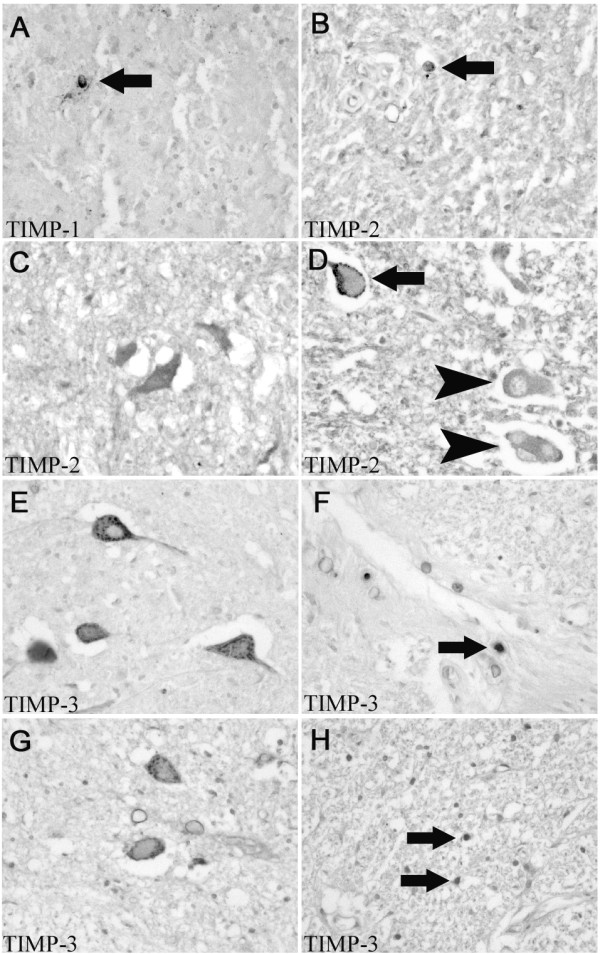
**TIMP-1, -2 and -3 in the human spinal cord after traumatic injury**. Images were taken from cases with different survival times after SCI in transverse sections. **A: **In a section from patients who died 2 days after SCI, immunohistochemistry for TIMP-1 demonstrated occasional cells (arrow) with a round to oval morphology at the lesion site. **B: **Immunohistochemistry for TIMP-2 showed an identical staining pattern, with single immunopositive rounded cells (arrow). **C: **At 4 days post injury, identifiable motoneurons demonstrated a cytoplasmic TIMP-2 immunoreactivity. **D: **8 days after SCI, only single neurons (arrow) were stained for TIMP-2 and many motoneurons were unstained (arrowheads). **E: **8 days after trauma, immunohistochemistry for TIMP-3 demonstrated cytoplasmic staining in most identifiable motoneurons close to the site of injury. **F: **In the same case, single TIMP-3 immunoreactive round cells could be seen (arrow). **G: **Four months after SCI, gray matter of the perilesional area showed mostly TIMP-3 immunoreactive motoneurons. **H: **Eight months after injury, multiple activated cells (arrows, presumably astrocytes) were immunopositive for TIMP-3 in areas directly surrounding the dense glial scar. **(A-H, mag. ×260)**

Only rare TIMP 1, 2 or 3 immunoreactive cells could be demonstrated at the lesion site up to 24 days post injury (Fig. 6A, B and 6F). These cells were rounded and were usually seen in the vicinity of blood vessels. By 4 months and longer, no specific TIMP-1 staining could be detected at or around the lesion site (not shown).

Immunohistochemistry for TIMP-2 demonstrated a temporal loss of neuronal staining. Immunoreactivity could be detected in most motoneurons and some interneurons for up to 4 days (Fig.6C). However, at survival times ranging from 8 to 24 days only single immunoreactive neurons were visible. This was particularly evident for TIMP-2 staining in motoneurons close to the lesion site (Fig.6D). From 4 months, the remaining neurons displayed a staining pattern comparable to the control cases with most distinguishable motoneurons being TIMP-2 positive and some positive interneurons in laminae I to VI (not shown). Apart from intermittent immunopositive macrophages and stained neurons, no other cell population demonstrated TIMP-2 immunoreactivity. In contrast to TIMP-2, the neuronal staining pattern of TIMP-3 remained unchanged over the range of survival times. About half of the motoneurons were stained and single interneurons could always be seen at and around the site of injury (Fig.6E and 6G). Activated astrocytes became transiently immunopositive for TIMP-3 between 8 months to 1 year after SCI (Fig.6H and 7G). Survival times of greater than 1 year only revealed neuronal TIMP-3 immunoreactivity (not shown).

## Discussion

MMPs in the CNS are largely synthesized by endothelial cells, microglia, astrocytes and neurons under normal conditions and can be produced at elevated levels in response to stress [9]. A number of reports have indicated that MMPs are involved in the pathogenesis of a wide range of diseases and disorders of the CNS, including neurodegenerative diseases such as Alzheimer's disease, amyotrophic lateral sclerosis, Parkinson's disease and progressive supranuclear cerebral palsy [10-14] as well as stroke injuries [15]. It has been suggested that this class of extracellular proteases may be appropriate molecular targets to reduce secondary tissue degeneration and improve functional outcome [7,16]. Although recent studies have demonstrated the involvement of MMPs in experimental models of spinal cord injury, there has, until now, been only one recent correlative investigation regarding the spatio-temporal distribution of MMP-9 in human spinal cord injury [17]. In the present study, the timing and distribution of MMP-1, -2, -9 and -12, as well as their tissue inhibitors TIMP-1, -2 and -3 were investigated in both normal and traumatically injured samples of human spinal cord. In an attempt to obtain tissue samples that were as comparable as possible, an emphasis has been placed on using samples obtained from cases which underwent similar lesion type and severity. Therefore, all patients with traumatic SCI suffered severe injuries of the maceration type (for a detailed description of the morphology of the lesion sites, see [18]) and were clinically diagnosed as having "complete" injuries. The paucity of human specimens led to the inclusion of cases from different age groups and varying levels of injury. Thus, the present data needs to be interpreted with care and can, by no means, be easily generalised to patients suffering from other types or severities of SCI. Thus, it is clear that future studies will be needed to verify if a similar pattern of post-traumatic MMP and TIMP expression at the lesion site can be found in less severe- as well as other types of SCI (e.g. following laceration and contusion type injuries). The comparison of the present data with previous results demonstrated that the spatio-temporal pattern of microglia/macrophages and astrocytic responses (i.e. the main cell populations demonstrating post traumatic MMP immunoreactivity in the present study) supports earlier investigations in human SCI [17].

### Expression of MMPs and TIMPs in the normal CNS

In the normal, unlesioned human brain and spinal cord, MMP and TIMP immunoreactivity was generally scarce. The data obtained from prior investigations on the expression of MMP-1, -2 and -9 in the human nervous system have been inconsistent, reporting either no immunoreactivity [17,19-21] or MMP-1 positive microglia [20], MMP-2 positive microglia, pericytes and blood vessels [7,14,22,23], MMP-9 immunoreactive neurons, microglia and single intravascular monocytes [10,22,23] and MMP-12 immunoreactive microglia and astrocytes [24]. No immunoreactivity for TIMP-1 and -3 has been detected. However, TIMP-2 has been found in endothelial cells and occasional neurons and astrocytes [20].

In the present investigation, neuronal staining for MMP-1, TIMP-2 and -3 was detected as well as individual MMP-9 positive intravascular monocytes. In general, the inevitable delays that occur before human *post mortem *tissues undergo fixation will result in sub-optimal antigen preservation. It therefore always remains possible that low levels of antigen, below the level of detection by the current immunohistochemical approach, may still be present at pathophysiologically relevant concentrations. All molecules were, nonetheless, clearly detectable in either both control or pathological cases.

Whereas the pattern of MMP-9 immunoreactivity observed in the present investigation supports pervious data [17], the neuronal expression of MMP-1 has, so far, not been reported. The expression of TIMP-2 and -3 in different populations of neurons, though not previously described in human material, is in line with animal data where both inhibitors were detected in cortical and cerebellar neurons [3].

The lack of immunoreactivity for MMP-2, -12 and TIMP-1 in the present control cases is unlikely to be attributable to a lack of sensitivity, since a clear signal could be detected in sections of traumatically injured spinal cord. It is possible, however remote, that the earlier described immunoreactivity in astrocytes, microglia and blood vessels may reflect more activated populations of cells in the previous control cases, despite the lack of morphological signs of disease. In contrast to an extensive literature on the function of MMPs and their inhibitors in pathological situations, there is hardly any information on their function under normal conditions apart from those instances where they have been associated with plasticity [4].

### Expression of MMPs and TIMPs following human spinal cord injury

In the present investigation, a post-traumatic up-regulation of MMP-1, -2, -9 and -12 was detected. Following experimental SCI, several studies have reported the involvement of various MMPs and TIMPs in the post-traumatic events at and around the lesion site. In particular, the temporal expression pattern of almost all MMPs has been studied after compression injury to the mouse spinal cord [6]. An up-regulation of multiple MMPs, including MMP-2, -9 and -12 was detected, which occurred in a time-dependent manner, i.e. an early elevation of one group of proteins including MMP-9 and a more delayed elevation of others including MMP-2 and -12. In contrast to the present investigation, MMP-1 could not be detected in mouse spinal cord samples, at least up to 5 days after injury [6].

The present *post mortem *investigation of human material revealed a lesion-induced bi-phasic pattern of raised MMP-1 levels at and around the lesion site. At survival times of up to 8 days, MMP-1 was expressed in macrophages and microglia within the lesion epicentre, however, at the later survival times of 4 months to 1 year, activated astrocytes at the border of the glial scar became strongly MMP-1 immunoreactive. The delayed up-regulation of MMP-1 in astrocytes in the spinal cord parenchyma around the lesion site might be a consequence of earlier pro-inflammatory cytokine production since *in vitro *investigations have demonstrated the release of MMP-1 from astrocytes when stimulated by TNF-alpha or IL-1β [25]. Furthermore, cytokines regulate the activity of both gelatinases A and B (MMP-2 and -9) in cultured rat astrocytes [25,26]. The early induction of MMP-1 in the present investigation may have been associated with the further pathological breakdown of the blood-spinal cord barrier (see later), however, the role of the later induction MMP-1 in reactive astroglia expression is less clear. Experimental studies on wound repair after skin lesions have demonstrated an increased expression of MMPs, including MMP-1 in the scarless healing process of fetal injuries [27,28]. Furthermore, an investigation into regeneration-associated factors in the adult rat optic nerve revealed an increased post-traumatic expression of MMP-1 in peri-lesional astrocytes [29]. Therefore, it might be possible that the presence of MMP-1 in the population of peri-lesional astrocytes at the border of the evolving glial scar, might play a role in limiting the extension of this residual tissue barrier.

Several reports have demonstrated post-traumatic and post-ischaemic increases in MMP-2 [6,30-32]. Increased MMP-2 signals were detected at both protein and mRNA levels, with elevated expression levels lasting several weeks following experimental rat spinal cord injury [32]. A recent investigation using MMP-2 knock-out mice demonstrated a more severe outcome following traumatic SCI in mice lacking MMP-2 [8]. Knock-out mice demonstrated a reduced white matter sparing, a more widespread reactive astrogliosis as well as an impairment in spontaneous locomotor recovery compared to wild-type littermates. In the wild-type animals undergoing SCI, MMP-2 was mainly expressed in astrocytes and some macrophages for up to 2 weeks at the borders of the lesion site. In the present human material, there was an early and brief induction of MMP-2 in macrophages and microglia which lasted from 2 to 8 days after injury. By 24 days, when the first clear morphological indication of scar formation was visible, MMP-2 expression was on the borderline of detectability. In contrast to the situation in experimental animals, it is likely that MMP-2 expression after human traumatic SCI is more involved with the early vascular and inflammatory events than with reactive astrocytosis. In contrast to SCI, previous studies using *post mortem *human tissue of multi-infarct induced dementia, ALS and Parkinson's disease demonstrated the expression of MMP-2 at the border of the evolving astroglial scar or in astroglia in the cerebral cortex or substantia nigra [12,13,30]. The cellular distribution of MMP-2 expression in human pathology therefore appears to differ significantly in relation to the particular disease or disorder under investigation.

Contused mouse spinal cord demonstrated substantial blood vessel wall and astrocytic MMP-9 immunoreactivity within 3 days of injury [7]. Furthermore, the use of MMP-9 knock-out mice following SCI demonstrated a favourable functional outcome compared to wild type animals, with reduced blood-spinal cord barrier permeability after the lesion and increased spared white matter [7]. In the present study of severe traumatic SCI of the maceration type of injury, MMP-9 was mostly expressed in macrophages/microglia, where levels rose progressively from 1 week to 3 weeks after injury. However, some neutrophils were also found to be MMP-9 immunopositive at survival times of up to 8 days after injury. Although delayed, this spatio-temporal expression pattern is similar to that observed in experimental investigations [6,31,32]. However, a recent investigation of human traumatic SCI, using a heterogeneous sample of patients, found an early increase in MMP-9 at the lesion site for up to 10 days after injury and showed neutrophils to be the only cellular source [17]. One possible explanation for the differences observed between the present investigation and that of Fleming and colleagues may be the differences in severity and mechanism of injury for the cases chosen. Therefore, additional studies which assess the influence of different mechanisms of injury (e.g. maceration or laceration) on the spatio-temporal patterns of cellular invasion and protein expression would be useful. This is of particular importance when experimental strategies are transferred to the clinical domain.

The up-regulation of MMP-12 following human SCI was delayed for up to 24 days, at which time an abrupt increase in the number of immunoreactive microglia/macrophages could be detected. Macrophages have already been described as a principal source of MMP-12 [33]. The use of PCR following experimental mouse SCI revealed that, of all MMPs induced following traumatic injury, the induction of MMP-12 was by far the most striking, being approximately 189-fold greater than the basal levels. Furthermore, the importance of MMP-12 expression was demonstrated by using knock-out mice, in which an improved functional outcome was observed following SCI. This beneficial effect was reported to be due to a reduced permeability of the blood-spinal cord barrier and hence reduced infiltration by neutrophils and macrophages, as well as a lack of direct MMP-12-induced toxicity [6].

In contrast to the MMPs investigated in the present study, the detection and distribution of their inhibitors TIMP-1, -2 and -3 was limited. Only occasional TIMP immuno-positive macrophages could be detected at survival times of 2 – 24 days post injury. Neuronal TIMP-2 immunoreactivity was qualitatively reduced in comparison to control cases. The imbalance between MMPs and their respective TIMPs in certain situations may contribute to the development of pathology. Such an imbalance has already been described following experimental SCI in mice and also in the cerebral cortex of patients suffering PSP [7,14]. The minor up-regulation of TIMPs detected in the present study is largely in line with experimental studies using mice, in which a short term, transient up-regulation of TIMP-1 was detected following spinal cord compression injuries [6]. Therefore, similar to animal investigations, the strong induction of multiple MMPs after human SCI was not accompanied by a concomitant expression of their inhibitors, allowing these proteins to exert their effects in the lesioned spinal cord. The only clear increase in TIMP immunoreactivity was detected for TIMP-3 at survival times of 8 months and 1 year. In sections from these cases, the same peri-lesional activated astrocytes which expressed MMP-1 were also immunoreactive for TIMP-3. It may be that the up-regulation of TIMP-3 acts to limit the effects of MMP-1, but this suggestion remains speculative.

## Conclusion

The involvement of MMP-1, -2, -9 and -12 has been demonstrated in the post-traumatic events after human SCI. This investigation comprised of 15 patients who died at a range of different survival times after trauma. Due to the difficulties in obtaining *post mortem *human tissue specimens, only one case could be studied at each survival time. Although cases with similar survival times demonstrated similar immunohistochemical staining patterns, the present results need to be interpreted with caution, and further studies with more human cases per survival time would be of significant value.

All 4 MMPs were mainly expressed during the first weeks after injury and are most likely involved in the destructive inflammatory events of protein breakdown and phagocytosis by infiltrating neutrophils and macrophages as well as enhanced permeability of the blood-spinal cord barrier. Their temporal expression pattern corresponds largely to prior experimental studies, several of which have indicated that inhibition of MMPs may lead to improved functional outcomes. However, the lack of any clear indication of the exact functional role (beneficial or detrimental) of many MMPs in human spinal cord, plus the lack of specific inhibitors suggests that more research on this issue is warranted. Nonetheless, the present data on the spatio-temporal expression of MMPs and TIMPs following human SCI has demonstrated that experimental animal models do not always accurately predict the timing and expression pattern of key molecules in the clinical situation, and that such correlative investigations are important for the logical extension of drug development from the laboratory to the clinic.

## Abbreviations

Blood Spinal Cord barrier – BSB

Central nervous system – CNS

Extracellular matrix – ECM

Glial fibrillary acidic protein – GFAP

Matrix Metalloproteinase – MMP

Spinal cord injury – SCI

Tissue inhibitors of metalloproteinases – TIMP

## Competing interests

The author(s) declare that they have no competing interests.

## Authors' contributions

AB designed and coordinated the study and drafted the manuscript. KP carried out the immunohistochemical stainings. BK participated in the design of the study, provided specimens and helped to draft the manuscript. DM participated in the design of the study, provided specimens and helped to draft the manuscript. JS participated in the design of the study, provided specimens and helped to draft the manuscript. JN participated in the design of the study and helped to draft the manuscript. GB participated in the design and coordination of the study and helped to draft the manuscript. All authors read and approved the final manuscript.

## Pre-publication history

The pre-publication history for this paper can be accessed here:


